# Value of infarct location in the prediction of functional outcome in patients with an anterior large vessel occlusion: results from the HERMES study

**DOI:** 10.1007/s00234-021-02784-x

**Published:** 2021-09-03

**Authors:** Manon L. Tolhuisen, Marielle Ernst, Anne M. M. Boers, Scott Brown, Ludo F. M. Beenen, Francis Guillemin, Yvo B. W. E. M. Roos, Jeffrey L. Saver, Robert van Oostenbrugge, Andrew M. Demchuck, Wim van Zwam, Tudor G. Jovin, Olvert A. Berkhemer, Keith W. Muir, Serge Bracard, Bruce C. V. Campbell, Aad van der Lugt, Phill White, Michael D. Hill, Diederik W. J. Dippel, Peter J. Mitchell, Mayank Goyal, Matthan W. A. Caan, Henk A. Marquering, Charles B. L. M. Majoie, on behalf of the HERMES collaborators

**Affiliations:** 1 Department of Biomedical Engineering and Physics, Amsterdam UMC, Location AMC, Amsterdam, The Netherlands; 2 Department of Radiology and Nuclear Medicine, Amsterdam UMC, Location AMC, Amsterdam, The Netherlands; 3 Department of Diagnostic and Interventional Neuroradiology, University Medical Center Göttingen, Göttingen, Germany; 4 Nico.Lab, www.nico-lab.com, Amsterdam, The Netherlands; 5 Altair Biostatistics, St Louis Park, MN USA; 6 CIC-Epidémiologie Clinique, 1433, Inserm, CHRU, Université de Lorraine, Nancy, France; 7 Department of Neurology, Amsterdam UMC, Location AMC, Amsterdam, The Netherlands; 8 Department of Neurology and Comprehensive Stroke Center, David Geffen School of Medicine, University of California, Los Angeles (UCLA), Los Angeles, CA USA; 9 Department of Neurology, Maastricht UMC, Maastricht, The Netherlands; 10 Cardiovascular Research Institute Maastricht, Maastricht, The Netherlands; 11 Department of Clinical Neurosciences, Department of Radiology and Hotchkiss Brain Institute, Cumming School of Medicine, University of Calgary, Calgary, Alberta Canada; 12 Department of Radiology, Maastricht UMC, Maastricht, The Netherlands; 13 Department of Neurology, Stroke Institute, University of Pittsburgh Medical Center, Pittsburgh, PA USA; 14 Department of Neurology, Erasmus MC University Medical Center, Rotterdam, The Netherlands; 15 Department of Radiology and Nuclear Medicine, Erasmus MC University Medical Center, Rotterdam, The Netherlands; 16 Institute of Neuroscience and Psychology, University of Glasgow, University Avenue, Glasgow, UK; 17 Department of Diagnostic and Interventional Neuroradiology, IADI, Inserm, CHRU, Université de Lorraine, Nancy, France; 18 Department of Medicine, University of Melbourne, Parkville, Victoria Australia; 19 Department of Neurology, Royal Melbourne Hospital, Parkville, Victoria Australia; 20 Translational and Clinical Research Institute, Faculty of Medical Sciences, Newcastle University, Newcastle upon Tyne, UK; 21 Department of Neuroradiology, Newcastle upon Tyne hospitals, Newcastle upon Tyne, UK; 22 Department of Community Health Sciences, O’Brien Institute for Public Health, University of Calgary, Calgary, Alberta Canada; 23 Department of Radiology, Cumming School of Medicine, University of Calgary & Foothills Medical Centre, Calgary, Alberta Canada; 24 Department of Medicine, Cumming School of Medicine, University of Calgary & Foothills Medical Centre, Calgary, Calgary Canada

**Keywords:** Acute ischemic stroke, Follow-up infarct location, Diffusion weighted imaging, Functional outcome

## Abstract

**Purpose:**

Follow-up infarct volume (FIV) is moderately associated with functional outcome. We hypothesized that accounting for infarct location would strengthen the association of FIV with functional outcome.

**Methods:**

We included 252 patients from the HERMES collaboration with follow-up diffusion weighted imaging. Patients received endovascular treatment combined with best medical management (*n* = 52%) versus best medical management alone (*n* = 48%). FIV was quantified in low, moderate and high modified Rankin Scale (mRS)-relevant regions. We used binary logistic regression to study the relation between the total, high, moderate or low mRS-relevant FIVs and favorable outcome (mRS < 2) after 90 days. The strength of association was evaluated using the c-statistic.

**Results:**

Small lesions only occupied high mRS-relevant brain regions. Lesions additionally occupied lower mRS-relevant brain regions if FIV expanded. Higher FIV was associated with a higher risk of unfavorable outcome, as were volumes of tissue with low, moderate and high mRS relevance. In multivariable modeling, only the volume of high mRS-relevant infarct was significantly associated with favorable outcome. The c-statistic was highest (0.76) for the models that included high mRS-relevant FIV or the combination of high, moderate and low mRS-relevant FIV but was not significantly different from the model that included only total FIV (0.75).

**Conclusion:**

This study confirms the association of FIV and unfavorable functional outcome but showed no strengthened association if lesion location was taken into account.

## Introduction 

Despite advances in treatment of acute ischemic stroke (AIS), many patients do not return to functional independence. Insight into the course of disease obtained by the prediction of functional outcome might help patient specific rehabilitation. For example, the patient and family could be informed on realistic expectations about recovery, and rehabilitation therapy could be focused specifically on the patient’s needs.

Follow-up infarct volume (FIV) is associated with functional outcome after AIS and has been suggested as a prognostic marker. However, FIV is only moderately associated with outcome: only 12% of functional outcome is explained by FIV [1]. Moreover, the association between the volume of infarcted tissue and functional outcome varies among lesion locations[2–4].

Infarcts that are located in highly modified Rankin Scale (mRS)-relevant regions negatively affect functional outcome, even when the lesion is small[2–5]. Ernst et al.[2] and Sheth et al. [6] showed that the association between lesion volume and functional outcome, measured by mRS at 90 days, is stronger if lesion location is taken into account. In the study of Ernst et al., an increase in lesion volume in high mRS-relevant areas was associated with a higher risk of unfavorable outcome. In their study, lesion volume was quantified on 3 to 9 days follow-up (FU) non-contrast computed tomography (NCCT) images.

Diffusion weighted imaging (DWI) is the preferred modality for assessing infarcted tissue due to its high sensitivity, which reaches near 100% sensitivity within 6 h after stroke onset[7, 8]. We therefore aimed to study whether the association between FIV as depicted on FU DWI and functional outcome according to the mRS at 90 days is strengthened when lesion location is taken into account for patients with an anterior large vessel occlusion.

## Materials and methods

### Patient population

We included patients from the HERMES collaboration with available FU DWI. The HERMES collaboration is a prospective meta-analysis of seven clinical randomized controlled trials (RCTs) that assessed the treatment efficacy of the combination of endovascular treatment (EVT) and best medical management including intravenous alteplase versus best medical management alone for patients with an occlusion within the proximal anterior circulation (ICA, M1 and M2 occlusions)[9]. In case patients were randomized for additional endovascular treatment, intravenous thrombolysis was administered within 4.5 h if eligible. Most trials allowed randomization for EVT within 6 h. The REVASCAT trial and the ESCAPE trial allowed randomization within 8 and 12 h respectively. Patients were excluded in case of poor FU DWI quality. Poor image quality scans included motion artefacts, noise or incomplete field of view. According to the trial protocols, if follow-up DWI was acquired, it was done at 24 h after treatment [10–14].

Each RCT in the HERMES collaboration was approved by the relevant national or local medical ethical committee. All medical images and reports were anonymized, and informed consent was obtained for each patient according to each trial protocol. Patients included in these trials consented for participation on the individual trials as well as additional research with the data.

### Lesion segmentation and regions

For segmentation, an initial coarse delineation was obtained by labeling all voxels as infarct positive that differed in intensity by ≥ 20% compared to the mirrored ROI at the contralateral side on trace DWI. Then, a subsequent manual adjustment was performed if needed by one of three expert neuroradiologists (WHvZ, LFMB or CM). Lesion segmentations also included areas with parenchymal hemorrhage within and adjacent to the infarct. In this study, hemorrhage was recognized on DWI as hypointense regions. If the patients received decompressive hemicraniectomy and no pre-surgical scan was available, only lesions within the theoretical boundary of the skull were included within the segmentation.

**Fig. 1:**
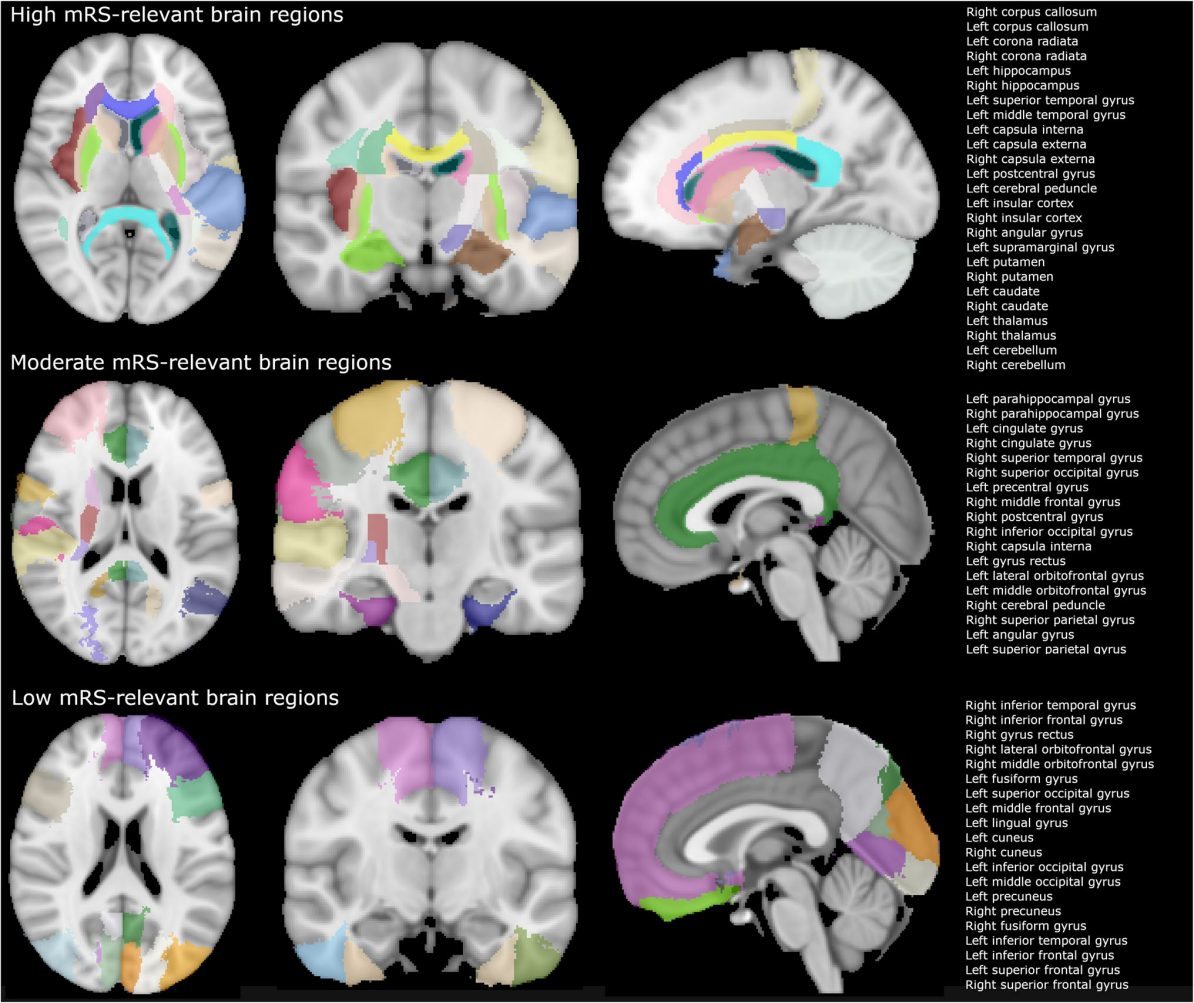
The division of brain regions according to high, moderate and low mRS relevance

Each brain was divided into 66 anatomical regions using three different atlases. We used the Laboratory of Neuro Imaging Probabilistic Brain Atlas [15], which includes 56 mostly cortical regions. These regions were extended with internal capsule, corona radiata, thalamus, corpus callosum and middle cerebral peduncles, which are part of the John Hopkins University International Consortium DTI-based white matter atlases [16] and Harvard–Oxford cortical and subcortical atlases [17–20]. The atlases were aligned to each DWI scan by affine and additional B-spline registration with the use of the statistical parametric mapping 8 toolbox (http://www.fil.ion.ucl.ac.uk/spm/). The regions were classified as high, moderate and low relevance for mRS, similar as presented by Ernst et al.[2] and according to a previously presented strength of association by Cheng et al. [3] (see Fig. 1). A voxel-based lesion mapping approach was used in which the impact of a brain region on functional outcome was represented by the Z-score acquired through a Brunner and Munzel Rank order test. Corresponding to these regions, total FIV was divided into sub-volumes categorized as being low, moderate and high mRS relevant.

### Statistical analysis

Medians and interquartile ranges (IQR) are reported for all continuous variables. Frequencies (*n*) and percentages (%) are reported for all categorical and dichotomous variables. Odds ratios are presented per 10 mL of FIV with 95% confidence interval. We tested for a significance level of *p* < 0.05.

Primary outcome was a favorable functional outcome according to the modified Rankin Scale (mRS), defined as mRS ≤ 2, at 90 days after stroke onset. For the primary outcome, we performed unadjusted univariable and multivariable logistic regression analyses. The independent variables for the univariable models were total FIV and high, moderate and low mRS-relevant FIV in milliliter. The independent variables for the multivariable model were high, moderate and low mRS-relevant FIV in milliliter. For each analysis, we performed an additional adjusted multivariable regression analysis in which we adjusted for age, sex, diabetes mellitus, atrial fibrillation, previous stroke or pre-stroke mRS > 0, treatment allocation, occlusion site, time from stroke onset to treatment and the collateral score.

Secondary outcome was shift (on the mRS) towards better functional outcome at 90 days, for which we performed ordinal logistic regression. We performed univariable and multivariable regression analysis as per the models for the primary outcome.

To study the quality of the statistical models, we computed the c-statistic and the Akaike information criterion (AIC). For logistic regression, the c-statistic is the area under the curve (AUC) of the receiver operating characteristic (ROC). An ROC graphically represents the ability of a binary classifier to predict the correct diagnosis as its discrimination threshold varies. The AUC represents the probability of the classifier to correctly predict the outcome. For ordinal outcomes, a single ROC no longer exists. In this case, the c-statistic can be computed from the cumulative ROCs [21]. We used DeLong’s test [21] to compare the different c-statistics. The AIC gives a measure of the relative quality of fit of the models: it tests how well a model fits the sampled data compared to the other models that were fitted on the same data.

## Results

**Fig. 2:**
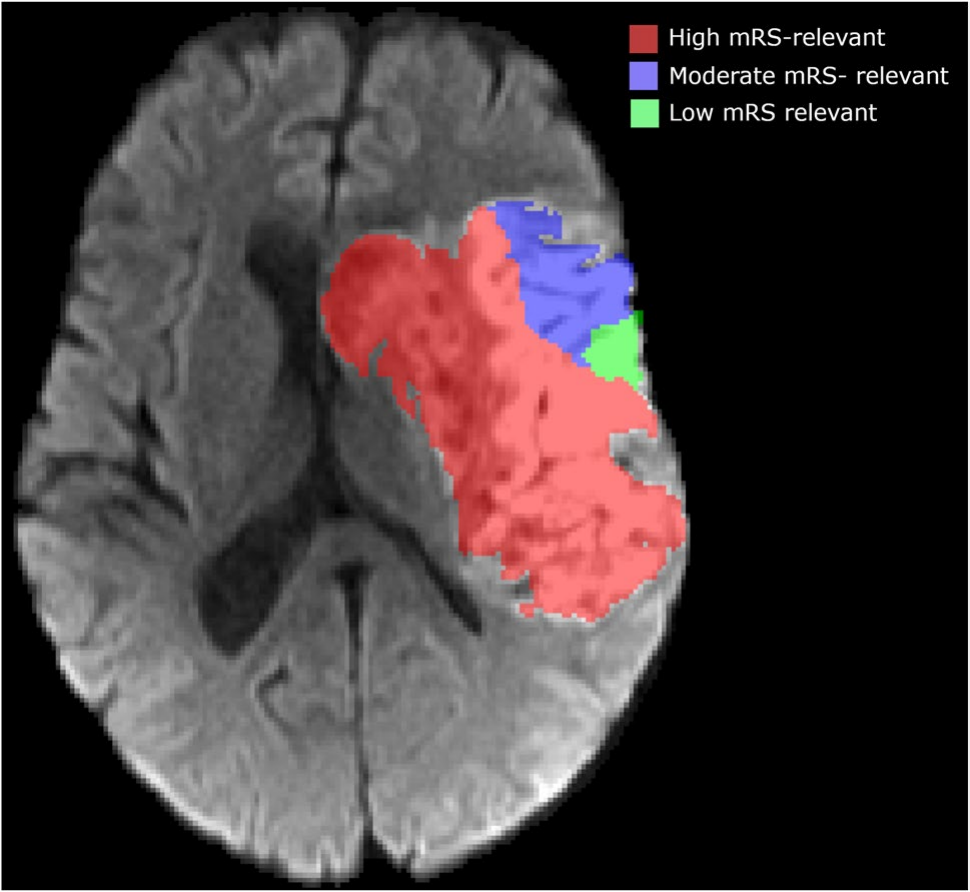
An example of the infarct segmentations on diffusion weighted MRI in areas with different mRS relevance

**Fig. 3:**
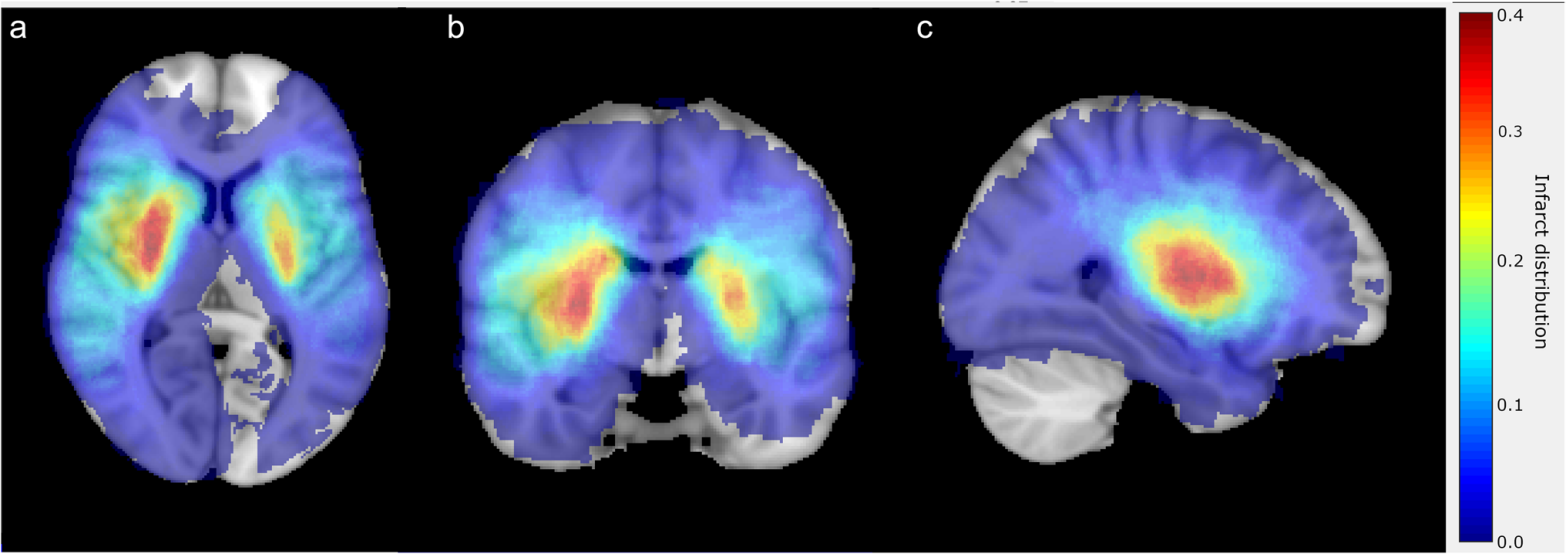
Infarct distribution shown for the axial (**a**), coronal (**b**) and sagittal (**c**) slice with the largest infarct probability present

Within the HERMES collaboration 307 patients had available 24-h FU DWI. We excluded 55 patients due to poor FU DWI quality, resulting in a total population of 252 patients. Baseline and follow-up characteristics for the subpopulation of this study and the overall HERMES patient group are shown in Table 1. The median age of the study population was 69 years, and a small majority was female. Most patients (81%) had a pre-stroke mRS of 0. The occlusion location was most common within the M1 segment (72%) of the MCA, followed by the ICA-T (22%). Only a small number of patients had a M2 occlusion (6.1%). Within our study population, 52% received endovascular treatment, from which 46% also received IVT and 46% received IVT alone. The remaining 2% received neither IVT nor EVT and supportive care only. Favorable outcome at 90 days was reached in 53% of the study population. Figure 2 shows an example of the infarct segmentations on diffusion weighted MRI in areas with different mRS relevance. Figure 3 shows the infarct distribution for the study population for the axial, coronal and sagittal slice with the largest infarct probability. Lesions were most often present in the right lentiform nucleus.

**Table 1:** Baseline and follow-up characteristics for our subpopulation and those of the HERMES dataset

Characteristic	Volume analysis subgroup (*n* = 252) Mean ± SD (*N*) [Median] (IQR) or % (*n*/*N*)	HERMES (*n* = 1764) Mean ± SD (*N*) [Median] (IQR) or % (*n*/*N*)
Age (years)	66 ± 14 (251) [69] (59, 76)	66 ± 14 (1761) [68] (57, 76)
Male gender	49% (124/252)	53% (929/1762)
NIHSS at baseline	17 ± 4.9 (251) [17] (13, 21)	17 ± 5.1 (1751) [17] (13, 21)
Diabetes mellitus	16% (40/251)	16% (287/1756)
Atrial fibrillation	44% (77/177)	33% (447/1351)
Prior stroke	11% (27/252)	11% (188/1751)
Pre-stroke mRS		
0	81.4% (144/177)	83% (1057/1280)
1	16% (28/177)	13% (162/1280)
2 +	2.8% (5/177)	4.8% (61/1280)
Occlusion location		
ICA-T	22% (55/246)	20.2% (350/1731)
M1	72% (176/246)	73.8% (1277/1731)
M2	6.1% (15/246)	6.0% (104/1731)
EVT allocation	52% (131/252)	49% (871/1764)
tPA delivered	93% (233/252)	89% (1572/1764)
Treatment		
EVT + tPA	46% (117/252)	43% (763/1764)
EVT only	5.6% (14/252)	6.1% (108/1764)
tPA only	46% (116/252)	46% (809/1764)
Best medical management	2.0% (5/252)	4.8% (84/1764)
Onset to randomization (min)	195 ± 97.6 (252) [180] (130.0,233.0)	202 ± 89 (1756) [183] (140.0,245.0)
NIHSS at baseline		
0–4	0.4% (1/251)	0.4% (7/1751)
5–15	42% (105/251)	37% (647/1751)
16–20	32% (81/251)	37% (655/1751)
21–42	26% (64/251)	25% (442/1751)
TICI 2b/3 (EVT-treated subjects only)	84% (97/115)	75% (550/729)
Favorable outcome at 90 days	53% (133/249)	47% (462/978)

**Fig. 4:**
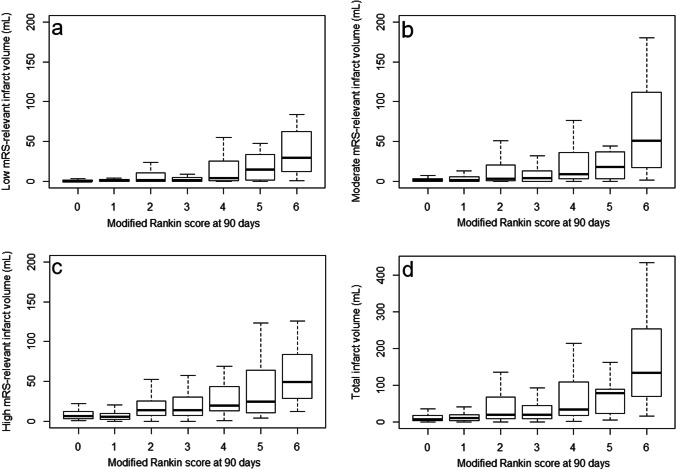
Box plots showing the infarct volume distribution per mRS category for the low (**a**), moderate (**b**) and high (**c**) mRS-relevant infarct regions and the total infarct volume (**d**)

Figure 4 shows box plots for the FIV for the low, moderate, high mRS-relevant regions and total FIV in relation to mRS with corresponding standard deviations. The increase of FIV was associated with a higher risk of unfavorable outcome for all the different regions. Moreover, the variance in infarct volume increased with worsening outcome, especially for high and low mRS-relevant regions.

**Fig. 5:**
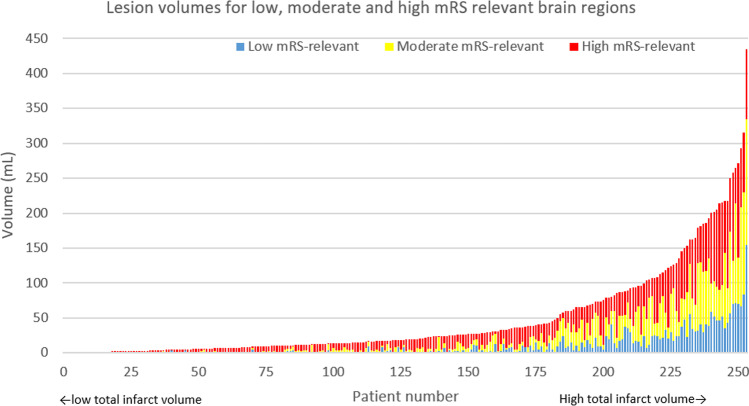
Lesion volume distribution classified as low, moderate and high mRS relevant. Total lesion volume increases along the x-axis. Each bar represents a single subject

Figure 5 shows the lesion volume for low, moderate and high mRS-relevant brain regions per patient. In patients with small total FIV, only high mRS-relevant brain regions were affected. With increasing total FIV, the lesions additionally occupied moderate mRS and subsequently low mRS-relevant brain regions.

Results for the logistic regression models and ordinal logistic regression are presented in Table 2. For the univariable logistic models (models 1 to 4), all volume measures were significantly associated with favorable mRS. The odds ratio was highest for total FIV and lowest for low mRS-relevant region FIV. For the multivariable model (model 5), only high mRS-relevant region FIV was significantly associated with favorable mRS. Similar results were obtained for the ordinal logistic regression model. Results after adjustments were comparable to the unadjusted analyses.

**Table 2:** Associations between the total follow-up infarct lesion volume and the follow-up infarct lesion volume divided in high, moderate and low mRS-relevant regions and favorable mRS at 90 days

		OR (95% CI)	AIC	c-stat	aOR (95% CI)†
Logistic regression (favorable outcome, mRS ≤ 2)					
Univariable					
Model 1	Total FIV	0.83 (0.77, 0.89)	300	0.75	0.78 (0.70, 0.86)
Model 2	High mRS-relevant FIV	0.62 (0.53, 0.74)	295	0.76	0.53 (0.42, 0.67)
Model 3	Moderate mRS-relevant FIV	0.72 (0.62, 0.83)	322	0.74	0.66 (0.54, 0.81)
Model 4	Low mRS-relevant FIV	0.50 (0.38, 0.66)	309	0.70	0.45 (0.31, 0.65)
Multivariable					
Model 5	High mRS-relevant FIV	0.67 (0.53, 0.84)	298	0.76	0.56 (0.41, 0.76)
	Moderate mRS-relevant FIV	1.00 (0.80, 1.25)			0.87 (0.66, 1.16)
	Low mRS-relevant FIV	0.86 (0.54, 1.35)			1.12 (0.65, 1.95)
Ordinal logistic regression (shift towards better outcome)					
Univariable					
Model 6	Total FIV	0.84 (0.81, 0.88)	830	0.72	0.83 (0.79, 0.87)
Model 7	High mRS-relevant FIV	0.70 (0.64, 0.77)	837	0.72	0.67 (0.60, 0.78)
Model 8	Moderate mRS-relevant FIV	0.72 (0.65, 0.80)	859	0.71	0.69 (0.61, 0.78)
Model 9	Low mRS-relevant FIV	0.52 (0.44, 0.62)	841	0.69	0.51 (0.42, 0.62)
Multivariable					
Model 10	High mRS-relevant FIV	0.79 (0.69, 0.91)	832	0.72	0.76 (0.65, 0.89)
	Moderate mRS-relevant FIV	0.89 (0.77, 1.04)			0.83 (0.67, 1.37)
	Low mRS-relevant FIV	0.86 (0.62, 1.21)			0.96 (0.67, 1.37)

†Adjusted for age, sex, diabetes mellitus, atrial fibrillation, previous stroke or pre-stroke mRS > 0, treatment allocation, occlusion site, time from stroke onset to treatment and the collateral score

For the univariable logistic regression models, the AIC was lowest for the model that included high mRS-relevant FIV. The model including high mRS-relevant FIV was a better fit to the data than the multivariable model. For the ordinal logistic regression models, AIC was lowest for the model that included total FIV.

Only small differences were seen in the c-statistic for the different models. For the logistic model, the c-statistic was highest for the model that included high mRS-relevant FIV. However, according to DeLong’s test, there was no significant difference between the c-statistic of the total FIV model (model 1) and the c-statistic for the high mRS-relevant FIV model (model 2). For the ordinal logistic regression models, the c-statistic was highest for both the model that included total FIV (model 6) and the high mRS-relevant FIV model (model 7).

## Discussion 

For patients with AIS caused by an anterior large vessel occlusion, our results did not show significant differences between models, and therefore no differences in strength of associations between infarct volume and outcome were observed when lesion location was taken into account.

Lesions within low mRS-relevant regions were only present in patients with higher total FIV. The results suggest that lesions progress from high mRS-relevant regions to less mRS-relevant regions when a stroke worsens. This agrees with the concept that leptomeningeal collaterals are relevant only to cortical MCA or ICA tissue and do not compensate for perfusion deficits in subcortical structures, such as the basal ganglia [22], and that ischemia progresses in case of late or inadequate reperfusion.

Our results did not correspond with the results presented by Ernst et al. [2], for which infarct lesion volume was quantified based on NCCT images. Their total FIV and high mRS-relevant FIV were overall larger compared to our study. Also, they showed a larger difference in volume between high mRS-relevant FIV and total FIV. The reason for discrepancy in our results was likely the difference in study populations. Ernst et al. only included patients from the MR CLEAN trial. The MR CLEAN trial included a relatively unselected group of patients with an anterior circulation large vessel occlusion (ICA, middle cerebral artery M1 and M2 segments, anterior cerebral artery A1 and A2 segments) within 6 h after stroke onset and a National Institutes of Health Stroke Scale (NIHSS) score above 2. No other imaging selection criteria as ASPECTS, collateral score or core and/or penumbra size on CTP were used. The inclusion criteria differed between trials and were more selective in some trials: patients were included if occlusions were present in only the intracranial carotid artery or middle cerebral artery (M1 segment) [13, 14, 23, 24], or only with more severe deficits [13], or with good collateral score [24], a core/penumbra mismatch[14] or smaller core [13] or a broader inclusion window [24].

Multiple studies have presented a voxel-based approach, studying the relation between specific anatomy and functional outcome [3, 4, 25–27]. For example, Cheng et al. [3] used voxel-based lesion symptom mapping to study the relation of infarct lesion measured on fluid-attenuated inversion recovery imaging and functional outcome. They presented statistical maps which show the relation between the spatial distribution of ischemic lesions and mRS at 1-month follow-up. Their results showed that the corona radiata, internal capsule and insula were of highest influence for mRS at 1 month. Laredo et al. [25] showed that large lesions strongly predict poor mRS, especially for insular lesions. Munsch et al. [4] studied the voxel-specific relation between infarct, measured on DWI, and mRS and cognitive function. Their results showed that infarct location is a significant predictor for cognitive function. However, they were not able to show a significant relation between location and dichotomous mRS. Wu et al.[5] showed that the posterior limb of the internal capsule, corona radiata and especially the white matter tracts were associated with greater severity of AIS and poor long-term outcome.

In the study population, lesions were most present in the right lentiform nucleus, which consist of the putamen and globus pallidus. These regions are responsible for the sensory and motor function, and learning processes. Russmann et al. [28] studied the effect of isolated lentiform nucleus infarcts. Infarcts in this region were related with sensory deficits, aphasia and hemineglect.

The current and previously presented moderate association of infarct volume and functional outcome is possibly explained by Goyal et al. in their recent publication [29]. They state that the variability of tissue vulnerability causes heterogeneity in ischemic tissue. More specific, even though tissue appears infarcted on imaging, it is possible that this tissue is still (partially) salvageable and recovers over time. The heterogeneity of ischemia has previously been studied in small studies. Nagesh et al.[30] showed in 9 patients that within 10 h after stroke onset ischemic lesions consisted of multiple heterogenic zones of ADC values. Guadagno et al.[31] showed, based on PET images of 5 patients, heterogeneity in blood flow and metabolism in regions that appeared hyperintense on DWI. Considering these insights, previously and current presented infarct volumes probably do not correctly represent true infarct.

This study suffers from some limitations. First, even though the HERMES collaboration combined multiple large randomized controlled trials with a heterogeneous population, our substudy population was relatively small and consists of highly selected patients. FU MRI is often not included within standard imaging protocol for FU stroke imaging. As a result, our study population only contained patients from the HERMES centers that included 24-h FU MRI within their study protocol. Moreover, the HERMES trials included only patients without prior disability, with proximal anterior circulation large vessel occlusions who were eligible for EVT. Since all patients had anterior circulation large vessel occlusions, the spectrum of patient deficits was more similar than occurs in an unselected stroke patient population that would include a majority with medium and small vessel occlusions in more locations, including patients with posterior circulation occlusions. Strategic location effects are likely to be more marked when overall lesion volumes are smaller and more varied. Also, our study population does not represent minor or severe cases of stroke. It is expected that patients with infarcts that affect low-mRS relevant regions experience fewer symptoms and are therefore less likely to present acutely in time window for acute interventions (IVT or EVT). Patients who did not meet the inclusion criteria for the trials due to contraindications, such as late presentation or high NIHSS score, were also not included. In addition, the majority of trials selected patients according to imaging characteristics such as the extent of changes on non-contrast CT or MRI, perfusion characteristics or collateral vessel quality. It is also plausible that MRI scans were only acquired for patients that had a better early outcome and were therefore able to tolerate MRI acquisition. Since patients with clinically or radiologically severe presentations are expected to have worse outcome than those included in the HERMES trials, future studies should assess the influence of lesion location and outcome for this population. Also, our study did not take hemispheric dominance into account. Finally, in this study, the mRS score was used for defining functional outcome. This score is a common endpoint in acute ischemic stroke trials and measures the degree of dependence in daily activities. The score is mainly focused on motor function (particularly walking) and is less sensitive for the evaluation of complex or cognitive functions such as memory or emotional processing [24]. Lack of association of radiologically defined tissue volumes weighted by relevance to the mRS therefore is strongly biased towards motor function and does not cover regions such as the cingulate gyrus (which is involved in emotional processing) or parahippocampal gyrus (which is involved in memory processing). In addition, simplifying the mRS outcome into an arbitrary dichotomy of “good” and “poor” may obscure structure–function relationships. Future studies should focus on the relation between the NIHSS in combination of the Montreal cognitive assessment score and infarct location. We expect a stronger relation between neurological impairment found with these scores and specific brain regions.

According to our results, information on the specific post-treatment infarct location depicted on DWI does not contribute to better estimation of treatment outcome. Probably, this is because the high mRS-relevant regions are always included within the infarct.

## Conclusion

Our results confirm the association between FIV as depicted on follow-up DWI and favorable functional outcome. We have shown that for patients with an infarct resulting from an ICA/MCA occlusion, the association between FIV quantified on follow-up DWI and functional outcome according to the modified Rankin scale (mRS) is not strengthened when lesion location is taken into account.
